# Identification and Functional Analysis of an Ammonium Transporter in *Streptococcus mutans*

**DOI:** 10.1371/journal.pone.0107569

**Published:** 2014-09-17

**Authors:** Arifah Chieko Ardin, Kazuyo Fujita, Kayoko Nagayama, Yukiko Takashima, Ryota Nomura, Kazuhiko Nakano, Takashi Ooshima, Michiyo Matsumoto-Nakano

**Affiliations:** 1 Department of Pediatric Dentistry, Osaka University Graduate School of Dentistry, Suita, Osaka, Japan; 2 Department of Pediatric Dentistry, Okayama University Graduate School of Medicine, Dentistry and Pharmaceutical Sciences, Okayama, Japan; LSU Health Sciences Center School of Dentistry, United States of America

## Abstract

*Streptococcus mutans*, a Gram-positive bacterium, is considered to be a major etiologic agent of human dental caries and reported to form biofilms known as dental plaque on tooth surfaces. This organism is also known to possess a large number of transport proteins in the cell membrane for export and import of molecules. Nitrogen is an essential nutrient for Gram-positive bacteria, though alternative sources such as ammonium can also be utilized. In order to obtain nitrogen for macromolecular synthesis, nitrogen-containing compounds must be transported into the cell. However, the ammonium transporter in *S. mutans* remains to be characterized. The present study focused on characterizing the ammonium transporter gene of *S. mutans* and its operon, while related regulatory genes were also analyzed. The SMU.1658 gene corresponding to *nrgA* in *S. mutans* is homologous to the ammonium transporter gene in *Bacillus subtilis* and SMU.1657, located upstream of the *nrgA* gene and predicted to be *glnB*, is a member of the PII protein family. Using a *nrgA*-deficient mutant strain (NRGD), we examined bacterial growth in the presence of ammonium, calcium chloride, and manganese sulfate. Fluorescent efflux assays were also performed to reveal export molecules associated with the ammonium transporter. The growth rate of NRGD was lower, while its fluorescent intensity was much higher as compared to the parental strain. In addition, confocal laser scanning microscopy revealed that the structure of biofilms formed by NRGD was drastically different than that of the parental strain. Furthermore, transcriptional analysis showed that the *nrgA* gene was co-transcribed with the *glnB* gene. These results suggest that the *nrgA* gene in *S. mutans* is essential for export of molecules and biofilm formation.

## Introduction

Membrane transporters are commonly found in living organisms and comprise one of the largest protein families, while their components are encoded by approximately 5% of the *Escherichia coli* and *Bacillus subtilis* genomes [1], [2]. Although these transporters are found in all species and are evolutionarily related, they are functionally diverse and participate in a wide range of important cellular functions. Bacterial transport systems enable bacteria to accumulate needed nutrients and extrude unwanted products, thus allowing bacteria to survive stress and create conditions condusive for growth and development [3]. Merrick et al. [4] noted that transport of ammonia across biological membranes is a key physiological process found in all domains of life. In addition, ammonium transporters have been described as important in supporting optimal growth rates for cells for ammonium uptake, especially when the concentration of NH3 is quite low [5], [6].

*Streptococcus mutans*, a Gram-positive facultative anaerobic bacterium, is considered to be a major etiologic agent of human dental caries and reported to form biofilms known as dental plaque on tooth surfaces [7]. This organism also possesses a large number of transport proteins in the cell membrane for export and import of molecules [8]. Nitrogen is an essential nutrient for Gram-positive bacteria, though alternative nitrogen sources such as ammonium can also be utilized [9]. Therefore, organisms have evolved highly effective systems for nitrogen acquisition and efficient utilization of scarce resources is ensured by a system of selective use of nitrogen sources [10]. In order to obtain nitrogen for macromolecular synthesis, nitrogen-containing compounds must be transported into the cell and, if necessary, degraded to either NH4^+^ or glutamate. The expression of enzymes required for the utilization of nitrogen-containing compounds is generally induced by their substrates. In addition, the expression of many degradative and transport systems is regulated in response to nitrogen availability in the growth medium [11]. Nitrogen metabolism in Gram-positive bacteria has been reported in a variety of studies [12]–[14] and demonstrated to be linked to virulence in *Staphylococcus aureus* [15]. However, the ammonium transporter (Amt) in *S. mutans* remains to be characterized.

Ammonium transport linked to nitrogen uptake is regulated via AmtB, a well-conserved ammonium transport membrane protein present in many bacterial species [12]. In *B*. *subtilis*, the NrgA protein encoded by the *nrgA* gene expresses the ammonium transporter, which is required for transport and utilization of ammonium at low concentrations [10]. Analysis of the complete genome of *S. mutans* strain UA159 in the Oralgen database (http://oralgen.lanl.gov/oralgen-tng/) indicates that the SMU.1658 gene corresponds to *nrgA* in *S. mutans*, functioning as the ammonium transporter, since it is homologous to the ammonium transporter gene in *B. subtilis*. In addition, regulation of nitrogen metabolism is coordinated by the PII type signal transduction proteins GlnB and GlnK, which control the activities of the membrane transport proteins and a transcription factor [16]. The PII protein family is composed of proteins that regulate enzyme activity, gene expression and are involved in nitrogen regulation, as well as glutamine synthesis activities in bacterial species [17], [18]. GlnK is homologous to GlnB and can substitute for GlnB to some degree [19]. In *S. mutans* UA159, SMU.1657 is located upstream of the *nrgA* gene and predicted to be *glnB*, a member of the PII protein family. The nitrogen regulatory proteins PII and AmtB are often paired and found in most bacteria [20].

For most bacteria, glutamine is an optimal source of nitrogen [21]. It is synthesized from ammonium, which is a major pathway for cells to assimilate the nitrogen required for biosynthesis of all amino acids, thus affecting protein synthesis and the structural and functional integrity of the cell [19]. *B. subtilis* uses alternative nitrogen sources such as ammonium, in the absence of glutamine. Ammonium utilization involves the uptake of the gas or the ammonium ion, the synthesis of glutamine by the glutamine synthetase and the recycling of the glutamate by the glutamate synthase [10].

*S. mutans* metabolizes carbohydrates to adhere to and form biofilms on tooth surfaces thus allowing the pathogen to tolerate rapid and frequent environmental fluctuations [22]. Oral biofilms are especially subject to a number of environmental fluctuations, such as nutrient availability, aerobic-to-anaerobic transitions, and pH changes [23]. Therefore, it is essential to study ammonium transporters, which play a crucial role in the uptake of nutrients by *S. mutans* in biofilms.

The present study focused on characterizing the ammonium transporter gene of *S. mutans* and its operon and regulatory genes were also analyzed. In addition, the influence of several inorganic nutrients on gene expression was examined.

## Materials and Methods

### Bacterial strains and culture conditions

*Streptococcus mutans* strain MT8148 (serotype *c*) was used in the present study [24]. Strain MT8148 was kindly provided by Professor Shigeyuki Hamada (Osaka University), whose group isolated *S. mutans* strains from Japanese children in the 1980's. We have used this strain as a reference strain in our laboratory for many years in a variety of experiments and published those results in several papers [25]–[30]. In addition, the director of the Ethic Committee of Okayama University Graduate School of Medicine, Dentistry and Pharmaceutical Sciences declared that approval from the ethic committee was not required for this study. *S. mutans* was grown in Brain Heart Infusion (BHI) medium (Becton Dickinson and Company (BDC), Franklin Lakes, NJ, USA) or Todd-Hewitt (TH) medium (BDC) as well as on Mitis-salivarius (MS) agar (BDC) at 37°C. When required, spectinomycin (SP; 1 mg/ml; Wako Pure Chemical Industries, Osaka, Japan) was supplemented.

*E*. *coli* XL-2 (Agilent Technologies, Santa Clara, CA, USA) and DH5α strains (Nippon Gene, Tokyo, Japan) were used as host strains for transformation of plasmid DNA. *E. coli* strains were grown in Luria-Bertani (LB; 1% tryptone, 0.5% yeast extract, 0.5% NaCl) medium while LB agar was prepared by the addition of 1.5% agar. When necessary, SP (100 µg/ml), Ampicillin sodium (AM; 100 µg/ml) and Tetracycline Hydrochloride (TC; 7.5 µg/ml) were added to the medium.

### Construction of a NrgA-deficient mutant

The procedure for generating the plasmid for construction of a NrgA-deficient mutant is described as follows. First, the internal DNA fragment of *nrgA* (approximately 500 bp at the upstream) was amplified by PCR with AmpliTaq (Life Technologies, Grand Island, NY, USA) using primer sets nrgAs-Eco-F and nrgAs-Bam-R (Table 1).The amplified DNA fragment was purified with phenol chloroform and precipitated with ethanol. This fragment was then ligated into a pGEM-T Easy Vector (Promega Co., Madison, WI, USA). The resultant plasmid was digested with *Eco*RI and *Bam*HI, and cloned into a streptococcal-*E. coli* shuttle vector which encodes for SP resistance (pSF152) to generate pCA01.

**Table 1:** Primers used in this study.

Names	Sequence (5′ to 3′)
nrgAs-Eco-F	GAA TCC GAG CTG ACC AAA TAA TCG T
nrgAs-Bam-R	GGA TCC GGC CTA CTC TGG TTT GGT T
nrgA-F	ATG GAT TCA GGA TCT ATA GCA TTT A
nrgA-R	CTA AGA ATC AAG TCC CAT AAA GGT T
nrgART-F	ATG GAT TCA GGA TCT ATA GC
nrgART-R	CCA ACA CCA CTA AAA GAT AAG G
SMU1656-F	GGC AAG ACT GCA GGA CCT GCA G
SMU1657-R	ACG GAG AAC GTG ATG GTG ATG CC
SMU1657-F	GAG CGT TGG AGT GAT CTT TTG ACC ACG G
SMU1658-R2	GAG AAG AAG CTA CTG GAC TTG ACG
SMU1658-F	CTT GCG GCG TCC CAA GCC TCC ATA G
SMU1659-R	GGG AGA ATG CCT CTT ACT GGT ATC TGG
SMU1657RT-F	GGC ATC ACC ATC ACG TTC TCC TGT ACG
SMU1657RT-R	CCG TGG TCA AAA GAT CAC TCC AAC GCT C

Transformation to *S. mutans* MT8148 was carried out with the protocol of Lindler and Macrina [31]. Overnight cultures of *S. mutans* MT8148 were inoculated into TH medium supplemented with 10% heat-inactivated horse serum (Invitrogen, Carlsbad, CA, USA) and incubated for 2 h. About 200 µg of pCA01 plasmid was added to growing liquid cultures and incubated for 2 h at 37°C. The cells were then collected by centrifugation, plated on MS agar containing SP (1 mg/ml) and incubated anaerobically at 37°C for 48 h. One positive transformant, NRGD, was selected and confirmed. Appropriate introduction of pCA01 into strain NRGD was confirmed by primer extension analysis. Following chromosomal DNA extraction of the transformants, primer extension analysis was used to determine the *nrgA* transcription sites in MT8148 with primers listed in Table 1 (nrgAs-Eco-F, nrgAs-Bam-R, nrgA-F and nrgA-R). Agarose gel electrophoresis of the PCR product showed an amplified band of approximately 500 bp. However, no extension product for NRGD was observed with the 1200 bp of nrgA-F and nrgA-R primers. To generate complemented strains (NRGD-comp), each mutant was transformed with plasmid pDL278 [32] containing the intact copy of the respective deleted gene.

### Bacterial growth rates

MT8148 and NRGD organisms were grown overnight at 37°C, then inoculated into TH medium or TH medium containing 20 or 40 mM ammonium. Growth curves were determined by measuring changes in optical density at 550 nm at 1-h intervals using a spectrophotometer (GE Healthcare, Fairfield, CT, USA). Furthermore, bacterial growth rates of NRGD were evaluated in the presence of 10 mM calcium chloride and 5 mM manganese sulfate, and 20 mM Urea. Three independent experiments were performed in triplicate.

### Biofilm assay

The ability of *S. mutans* strains to form biofilms was assessed by growing cells in wells of 96-well polystyrene microtiter plates (BDC). TH medium (diluted 1∶4) containing 0.1% sucrose was added with 1 µl of pregrown cell suspension, and then 100 µl of the prepared samples were inoculated into the individual wells, using ten wells per strain. The plates were incubated at 37°C with 5% CO_2_ for 48 h. After incubation, formed biofilms were stained with 1% crystal violet (Sigma-Aldrich, St. Louis, MO, USA) for 15 min at room temperature. The plate was next rinsed 6 times with sterile distilled water to remove loosely bound bacteria and any crystal violet that was not specifically staining the adherent bacteria. The washed plate was inverted several times on a Kimtowel paper towel (Kimberly-Clark, Irving, TX, USA) to dry and fixed with 95% ethanol. Finally, the plate was air dried and biofilms solubilized with MilliQ. Stained biofilms were quantified by measuring the absorbance at 570 nm with an enzyme-linked immunosorbent assay microplate reader (Thermo Fisher Scientific, Waltham, MA, USA). Three independent experiments were performed in triplicate.

### Confocal laser scanning microscope (CLSM) observation of biofilms

Quantitative and structural analysis of biofilms by confocal laser scanning microscopy was assayed according to the method described by Kuboniwa *et al*. [33]. MT8148 and NRGD were cultured in THB with 10 mM, and 20 mM glutamine overnight. Following incubation, strains were centrifuged and washed with distilled water. Next, the bacterial cells were labeled with 5 µl of 10 mM hexidium iodide (Invitrogen) and incubated in the dark for 15 min at room temperature. Each cell suspension was adjusted to 0.1 at an optical density of 600 in a chemically defined medium supplemented with 0.5% sucrose (sCDM) [34]. The saliva specimens were collected from two of the authors (KN and TO) of the present study, thus the need for Ethics Committee of Okayama University Graduate School of Medicine, Dentistry and Pharmaceutical Sciences approval was waived. The saliva was diluted 1∶4 with MilliQ to produce 25% saliva. Biofilms were formed in Lab-Tek Chambered #1.0 Borosilicate Coverglass System 8 chamber (Nunc, Rochester, NY, USA) that were coated with filtered 25% human saliva. The chamber was then incubated at 37°C with light shielding in an anaerobic chamber for 24 h. At the end of the experimental period, the sCDM was removed and PBS was added.

Imaging was performed using confocal laser scanning microscopy LSM 510 (Version 4.2, Carl Zeiss MicroImaging Co., Ltd., Jena, Germany) with a laser wavelength of 543 nm and the biofilm images of each sample were acquired from three random positions. The confocal images were analyzed by Image J for Macintosh (Version 10.2, Bethesda, MD, USA).

### Fluorescence efflux measurement

Fluorescence measurements were performed by a modification of methods described by Ocaktan *et al*. [35] with some modifications. The MT8148, NRGD strains, and NRGD-comp strains were grown until 0.4 at an optical density of 550 nm in TH medium and pelleted by centrifugation at 2400 *g* for 10 minutes at 4°C. The cells were then washed with 10 mM NaCl-50 mM NaPB (pH 7.0) and suspended again in the same buffer. Prior to fluorescence probe labeling, cultures were adjusted to an optical density of 0.2 at 600 nm and 1 ml of the adjusted samples was transferred to 13×100 mm test tubes (IWAKI, Shizuoka, Japan).

The adjusted samples were then labeled with fluorescence probe (1-(4-trimethylammoniumphenyl)-6-phenyl-1,3,5-hexatriene *p*-toluenesulfonate), (TMA-DPH; Invitrogen) [36], reacted at final concentrations of 1 µg/ml and 2 µg/ml and incubated with light shielding for 30 min. Following incubation, labeled cultures was centrifuged at 2400 *g* for 10 min at 4°C and the resulting pellets were washed twice with 500 µl of 10 mM NaCl-50 mM NaPB (pH 7.0). Thereafter, 100 µl samples were plated in 96 well plates (Nunc, Roskilde, Denmark) and absorbance was measured with a Twinkle LB970 fluorometer (Berthold Technologies GmbH & Co. KG, Bad Wildbad, Germany) with wavelengths at 355/460 nm.

### Northern blot hybridization

Total RNA was purified as previously described [37]. Briefly, overnight culture of MT8148 were added to TH medium and grown to late exponential phase at an optical density of 600 nm. The cells were collected by centrifugation at 2,400×*g* for 15 min at 4°C and suspended in diethyl pyrocarbonate (DEPC-treated water). The mixtures were transferred to Lysing matrix B (MP Biomedicals, Santa Anna, CA, USA) and isolated with TRI reagents (Sigma-Aldrich). Chloroform (200 µl) was then added to the RNA and vortexed. Next, RNA was resuspended in 500 µl chloroform, precipitated with isopropanol and washed two times with 75% ethanol. The resulting pellets were dried and suspended in DEPC treated water. RNA samples were then treated for 15 min at 37°C with RNase-free DNase (Promega). RNA (5 µg/ml) was then added to a loading buffer incubated at 65°C for 10 min and immediately placed on ice for 1 min. RNA was next loaded onto a formaldehyde gel and transferred to positively charged nylon membranes (GE Healthcare).

To prepare the probe, 200 bp fragments of the *nrgA* gene were PCR amplified using primers nrgART-F and nrgART-R (Table 1) and labeled according to the DIG high prime DNA northern labeling kit (Roche).

RNA fixed to the membrane was pre-hybridized with DIG easy hybridization solution (Roche) at 50°C for 30 min, followed by DNA probe hybridization with rotation and gentle agitation overnight. Subsequently, the membrane was incubated for 30 min in blocking solution and 30 min in antibody solution (75 mU/ml antidigoxigenin-AP). Following incubation, the membrane was rinsed twice times with washing buffer for 15 min, equilibrated with detection buffer for 3 min, and finally the membrane was incubated with CDP-*star* (Roche) and exposed to x-ray film for 20 min.

### PCR analysis of the *nrgA* operon and adjacent genes

To characterize the *nrgA* operon, RNA was extracted from cells grown to late exponential phase as described above. The RNA samples were treated for 15 min at 37°C with RNase-free DNase (Promega). SuperScript III Reverse transcriptase (Invitrogen) and random primers (Promega) were used to obtain complementary DNA (cDNA) from DNA-free RNA. PCR was then performed on DNA (as a positive control), cDNA and MilliQ (as a negative control), with specific primers that span *serC* and *glnB* (SMU1656F and SMU1657R), *glnB* and *nrgA* (SMU1657F and SMU1658R2), as well as *nrgA* and SMU.1659 (SMU1658F and SMU1659R) (Table 1).

### Quantitative real-time PCR

The quantitative real-time reverse transcription-PCR (qRT-PCR) was performed to evaluate the expression of the *nrgA* and *glnB* genes. Real-time RT-PCR was performed using complementary DNA samples with either 16S ribosomal RNA (rRNA) or specific primers. The expression of *glnB* gene with SMU1657RT-F and SMU1657RT-R primers was determined in MT8148 and NRGD (Table 1). Moreover, primers nrgART-F and nrgART-R and SMU1657RT-F and SMU1657RT-R were used to monitor *nrgA* and *glnB* gene expression under the influence of 2 mM glutamine (Table 1). The qRT-PCR reaction was conducted with SYBR green (Biorad) and run in an iCycler thermal cycler (Biorad), according to the manufacturer's instructions.

### Statistical analysis

All quantitative data are expressed as means ± SD of at least three independent experiments. Statistical analysis of variance (ANOVA) was employed to compare mean values, and *P* values<0.05 indicate statistically significant differences.

## Results

### Inactivation of *nrgA* affects the bacterial growth rates

**Figure 1:**
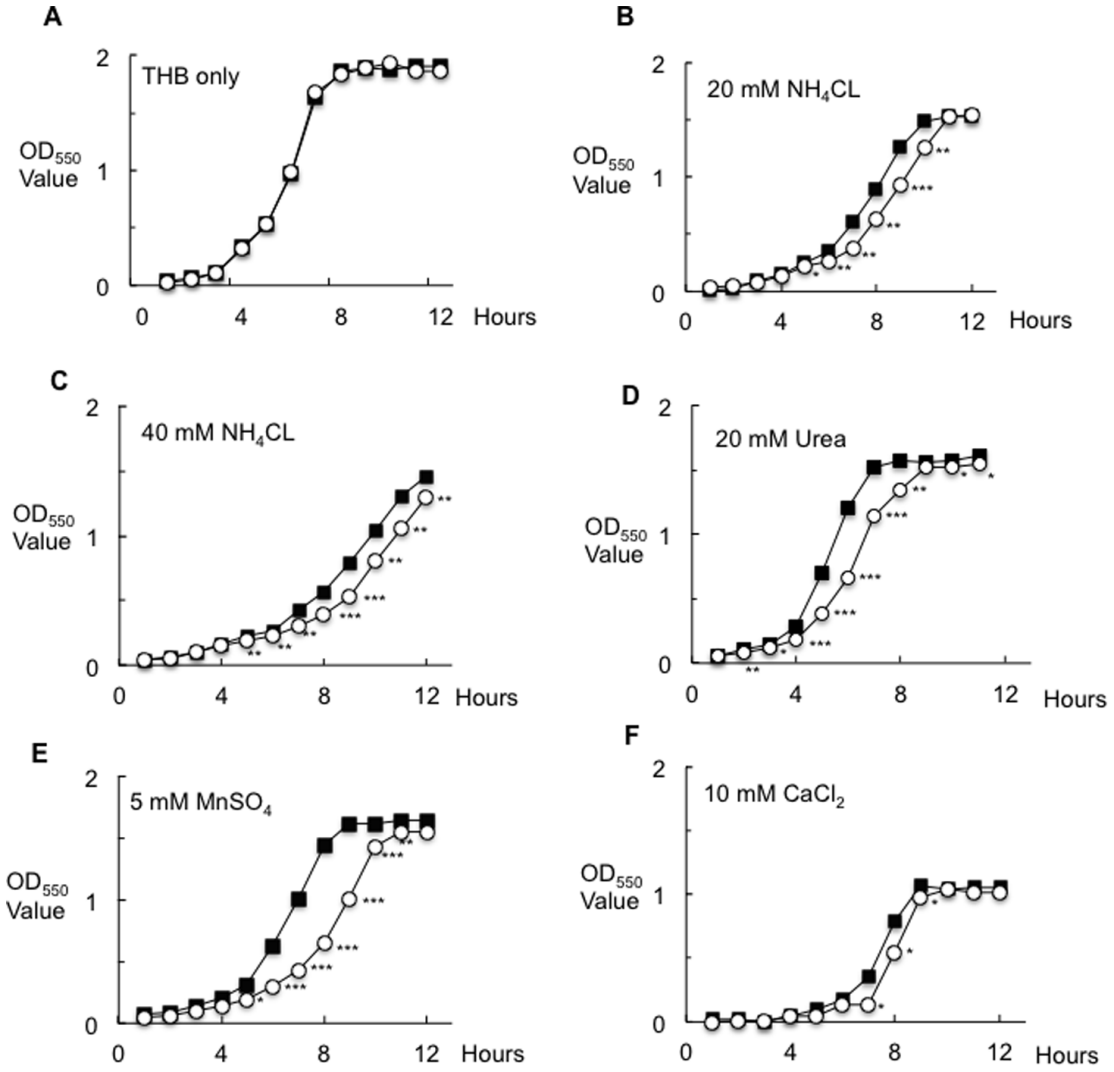
Bacterial growth rates of MT8148 and NRGD. **A.** THB only. **B.** THB with 20 mM ammonium chloride. **C.** THB with 40 mM ammonium chloride. **D.** THB with 20 mM Urea. **E.** THB with 10 mM calcium chloride. **F.** THB with 5 mM manganese sulfate. ▪ MT8148, ○ NRGD. Three independent experiments were performed in triplicate. There were significant differences in the values between MT8148 and NRGD (*P<0.05, **P<0.01, and ***P<0.001, ANOVA).

Figure 1A shows the bacterial growth curves of MT8148 and NRGD when cultured in THB alone. There were no significant differences in growth rates between these strains. On the other hand, the NRGD strain grown in the presence of 20 mM ammonium chloride showed significantly decreased growth as compared to MT8148 from 5 to 10 hours (Fig. 1B). In the presence of 40 mM ammonium chloride, NRGD also grew poorly and a significant difference was identified after 5 hours (Fig. 1C). In the presence of 20 mM urea, the growth rate of NRGD was also delayed, and significant differences were detected between 2 to 8 hours (Fig. 1D). In the presence of 10 mM calcium chloride and 5 mM manganese sulfate, the growth rate of NRGD was also delayed, and significant differences were identified between the strains in the periods from 7–9 hours and 5–11 hours respectively (Fig. 1E and F). This suggested that the ammonium transporter in *S. mutans* is important for not only uptake of ammonium but also other inorganic metabolites as nutrients.

In the presence of 20 mM ammonium chloride, the growth of NRGD was slightly changed as compared to that of the wild-type at pH 7.0, while that of NRGD was clearly delayed as compared to that of the wild-type at pH 5.0 (Fig. S1). In addition, in the presence of 20 mM urea, the growth of NRGD was delayed as compared to the wild-type. However, increasing the concentration to 40 mM Urea restored the growth of NRGD relative to the wild type (Fig. S2).

### Biomass and structure of biofilms

**Figure 2:**
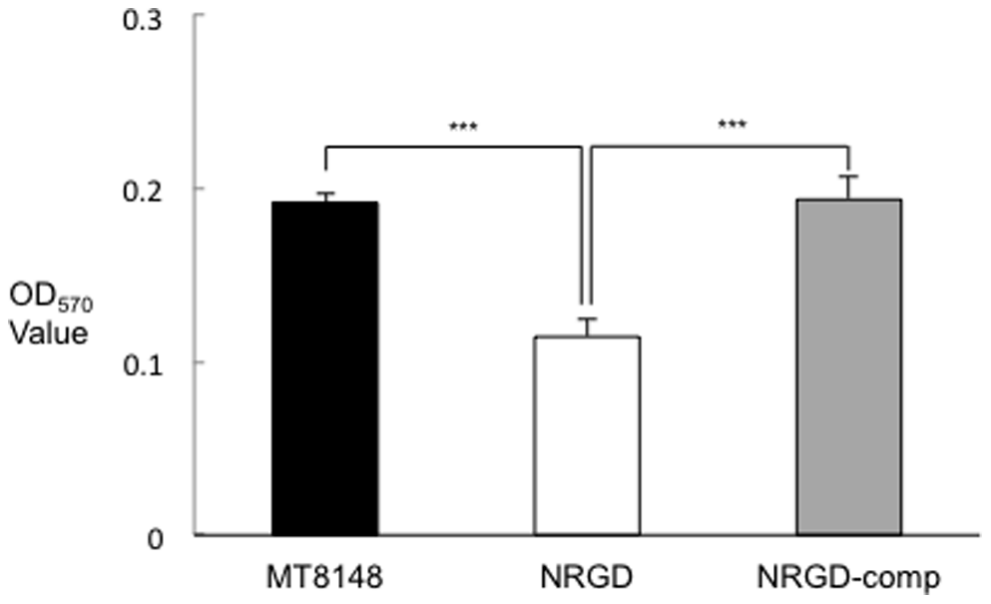
Quantity of biofilms formed by MT8148, NRGD, and NRGD-comp in 1:4 diluted THB containing 0.1% sucrose. Three independent experiments were performed in triplicate. There were statistically significant differences in the quantity of biofilms formed among these strains (***P<0.001, ANOVA).

**Figure 3:**
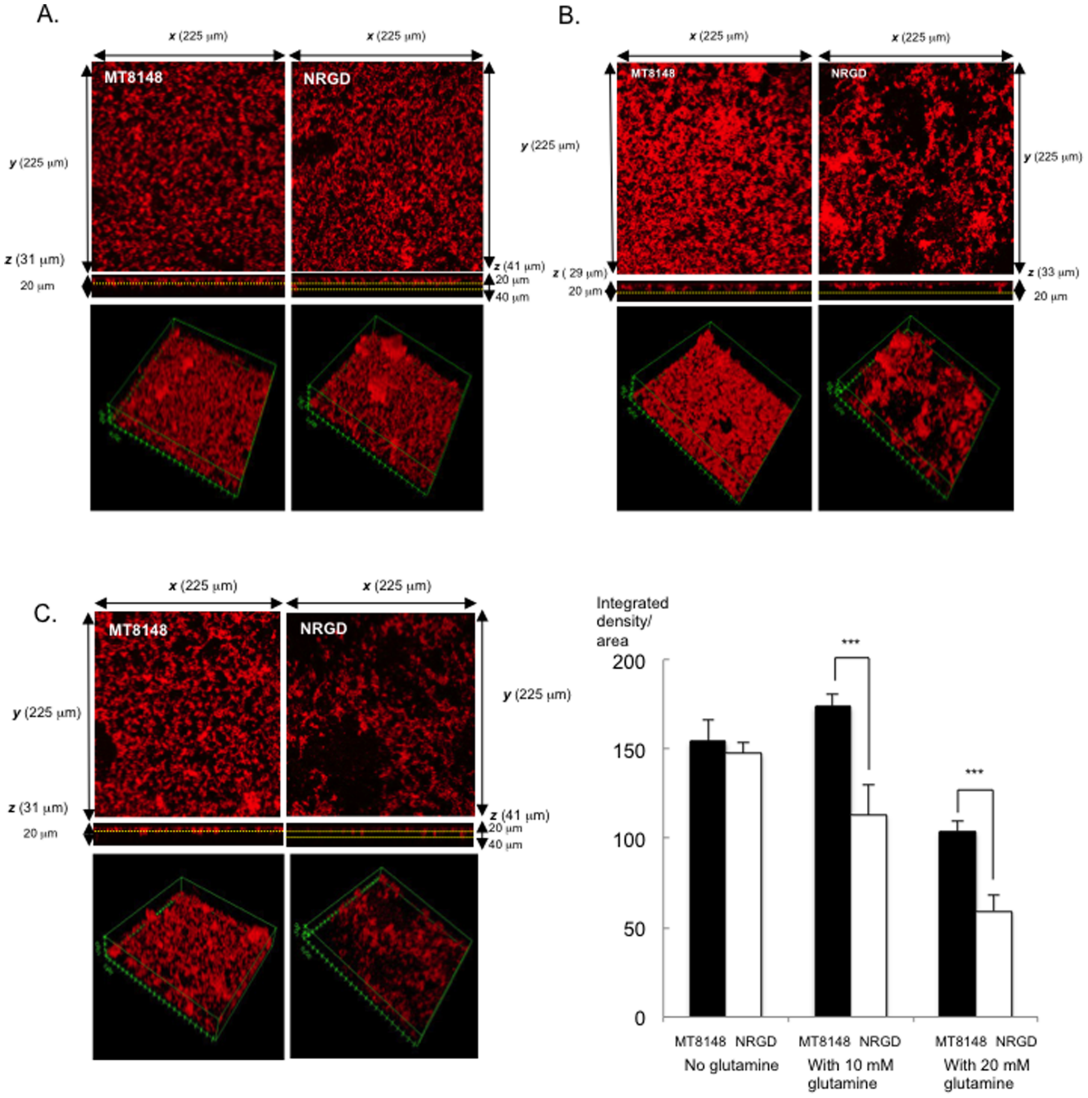
CLSM images of MT8148 and NRGD. **A.** MT8148 and NRGD grown in medium. **B.** MT8148 and NRGD grown in medium supplemented with 10 mM glutamine. **C.** MT8148 and NRGD grown in medium supplemented with 20 mM glutamine. **D.** Analysis of the densities of biofilms generated by MT8148 and NRGD with or without glutamine. The biofilm images of each sample were acquired from three random three positions. There was a statistically significant difference between MT1848 and NRGD (***P<0.001, ANOVA).

Biofilms formed by NRGD displayed significantly lower quantities than those formed by MT8148 (Fig. 2). Most importantly, biofilm formation was completely restored in the complemented strain. Confocal laser scanning microscopy (CLSM) with hexidium iodide staining was also performed to examine *S. mutans* biofilms attached to the wells of polystyrene plates. The advantage of using a nucleic acid stain such as hexidium iodide in biofilm studies is to maintain sufficient intensity for visualization with confocal microscopy with minimum toxicity as well as loss of cell viability [38]. According to the evaluation with CLSM images, biofilms formed by MT8148 had greater thickness than those formed by NRGD, while NRGD biofilms showed both small and large amorphous micro-colonies (Fig. 3A).

Three-dimensional images of the biofilms revealed that addition of 10 mM glutamine gave rise to thicker biofilms formed by MT8148 (Fig. 3B). In contrast, such addition resulted in a significant loss of micro-colony formation and more coarse structure in NRGD biofilms as compared to those formed by MT8148. Finally, the biofilm mass formed in the presence of 10 mM glutamine by NRGD was drastically decreased as compared to that formed by MT8148, which was supported by the results of quantitative assays (Fig. 3D). On the other hand, the images of the biofilms revealed that addition of 20 mM glutamine decreased the thickness of biofilms formed by MT8148 as compared to those formed by the addition 10 mM glutamine, while such addition resulted in a significant loss of micro-colony formation and more coarse structure in NRGD biofilms as compared to those formed by the addition 10 mM glutamine (Fig. 3C). That increased glutamine was affected the biofilm formation, which was supported by the biomass assay (Fig. 3D).

### Analysis of exocytosis via NrgA

**Figure 4:**
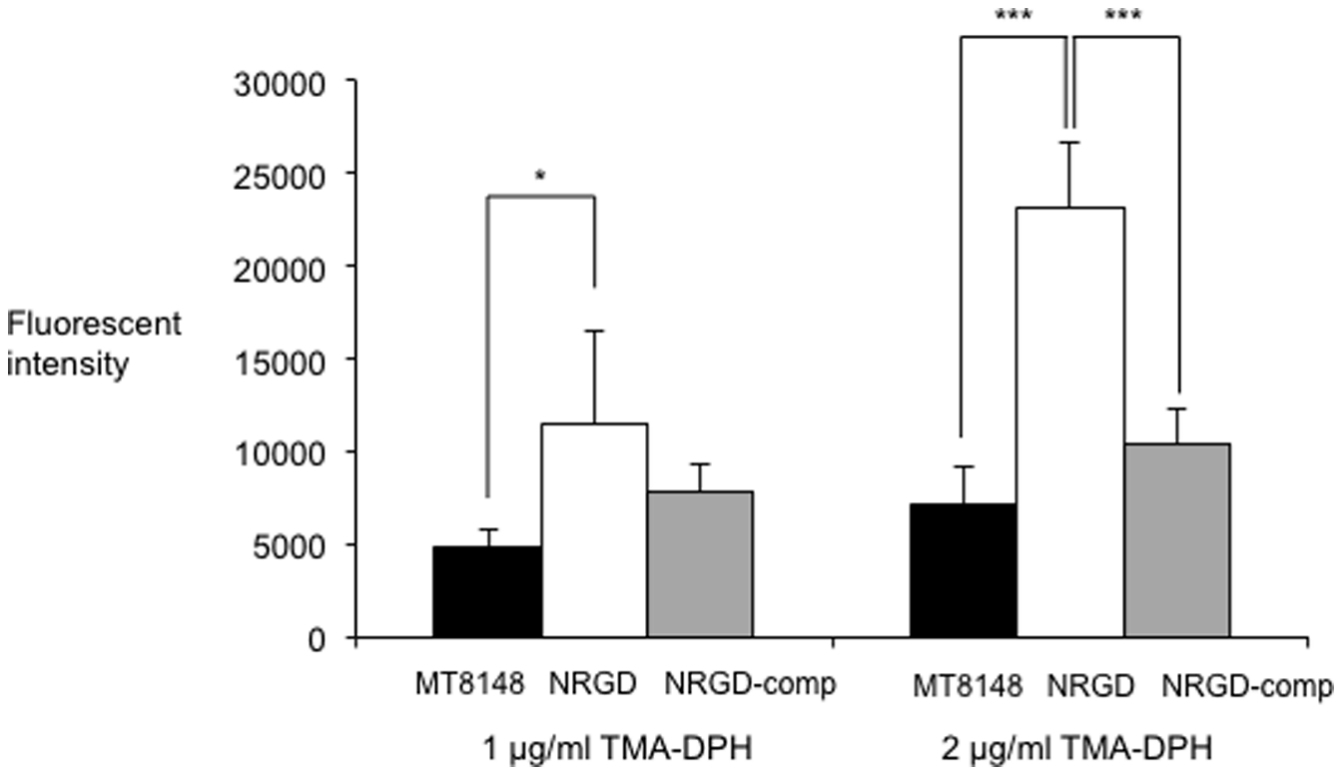
Analysis of exocytosis of MT8148, NRGD, and NRGD-comp with TMA-DPH. Fluorescence of the cells was determined in the presence of different concentrations of fluorescent probe, TMA-DPH. Three independent experiments were performed in triplicate. There was a statistically significant difference among strains (*P<0.05 and ***P<0.001, ANOVA).

Addition of 1 and 2 µg/ml of TMA-DPH led to an increase in the fluorescence intensity of MT8148, NRGD, and NRGD-comp at 355/460 nm (Fig. 4). However, the intensity of NRGD was significantly greater as compared to that of MT8148, suggesting a decrease in the amount of molecules released. In addition, the wild-type phenotype was completely restored in the complemented strain. Fluorescent probes are suitable for uptake experiments because they are non-fluorescent in aqueous environments, while they become strongly fluorescent in nonpolar or hydrophobic environments [35]. TMA-DPH is a fluorescence polarization probe known to be sensitive to plasma membrane surfaces [39]. The present analysis of exocytosis with TMA-DPH showed a decreased amount of molecules released from the ammonium transporter in the plasma membrane. Based on the permease type and energy source, Saier [40] characterized the ammonium transporter as a carrier type that functions in the efflux of ions, molecules, and toxic substances. This is consistent with the present findings, as it was found that inactivation of the *nrgA* gene blocked the export functions of *S. mutans*. Therefore, it seems that the *nrgA* gene has a function related to export of molecules, which may be one of the strategies used by *S. mutans* to respond to changes in its environment.

### Evaluation of *nrgA* and its operon

Northern blot analysis of the transcriptional organization of the *nrgA* gene locus detected a band specific to the *nrgA* and *glnB* genes, which was estimated to be approximately 1600 bp (data not shown). The molecular size of the band was found to be consistent with the 1578-bp band that spans the *nrgA* and *glnB* genes determined from the nucleotide sequence of this region.

**Figure 5:**
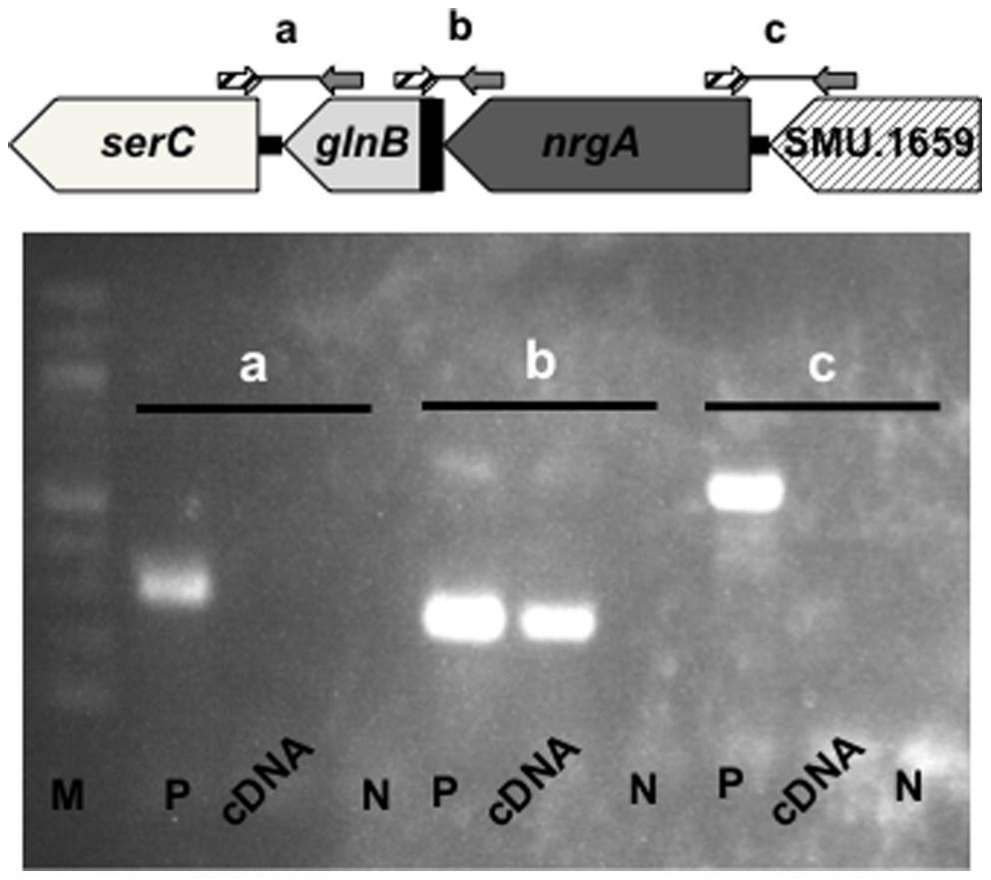
Evaluation of *nrgA* and its operon. PCR analysis of the organization of the *nrgA* operon and adjacent genes by using cDNA. The letters a, b, and c correspond to the amplified regions illustrated above the electrophoresis gel. Lanes: M, 100 bp DNA Ladder; P, chromosomal DNA of MT8148; cDNA, cDNA of MT8148, N, MilliQ.

Transcriptional analysis using cDNA with specific primers showed that primer extension yielded an amplified band indicating that *glnB* and *nrgA* constitute an operon. On the other hand, no amplified bands were detected in the A and C regions with use of cDNA, suggesting that *serC* and *SMu1511* are not part of the same operon as *glnB* and *nrgA* (Fig. 5).

**Figure 6:**
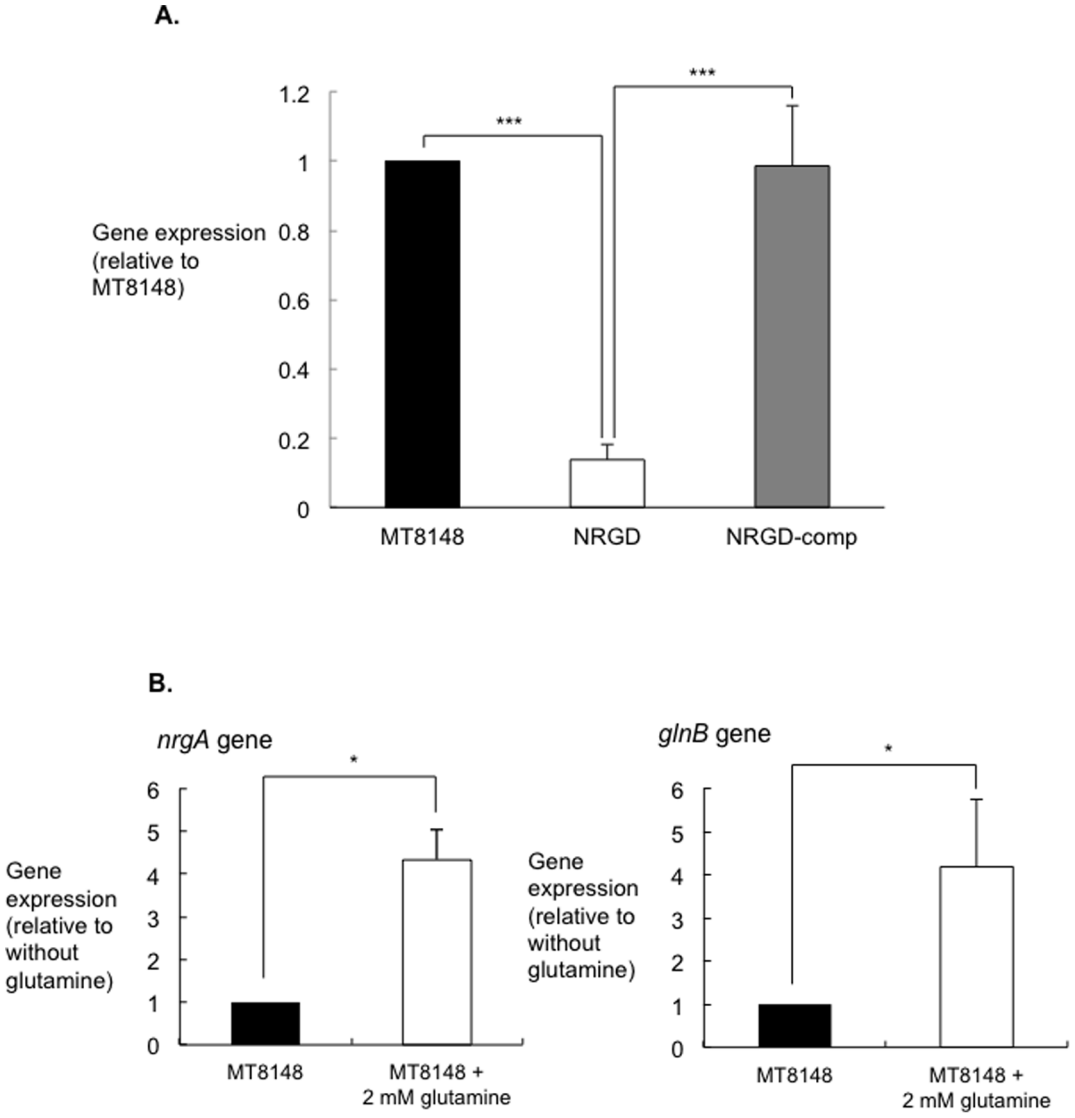
Evaluation of *glnB* and *nrgA* expression. **A.** Expression levels of *glnB* in MT8148, NRGD, and NRGD-comp. Transcript levels were measured using real-time PCR with 16SrRNA as the control. There was a statistically significant difference among these strains (***P<0.001, ANOVA). **B.** Real-time quantitative RT-PCR analysis of the influence of glutamine on *nrgA* and *glnB* gene expression. There were statistically significant differences in the expressions of the *nrgA* and *glnB* genes with and without glutamine (*P<0.05, ANOVA).

Real-time RT-PCR demonstrated that *glnB* gene expression in NRGD was drastically decreased as compared with that in MT8148 (Fig. 6A). In addition, the wild-type phenotype was completely restored in the complemented strain. In the presence of 2 mM glutamine, real-time RT-PCR assays showed that the *nrgA* and *glnB* genes were elevated as compared to in its absence (Fig. 6B). The PII protein, a product of the *glnB* gene, plays a central role in signal transduction of nitrogen-regulatory systems in prokaryotes and controls transcription under conditions of nitrogen limitation [41], [42]. In addition, the present results also demonstrated that the PII protein encoded by *glnB* regulates expression of the putative ammonium transporter.

## Discussion

For successful colonization and biofilm formation, *S. mutans* has developed multiple strategies [43], which help the pathogen to grow under nutrition-limiting conditions and protect it from various environmental insults [44]. The transport systems are one of the methods used for survival and *S. mutans* contains more than 280 genes associated with various transport systems involved in uptake of ions, molecules and carbohydrates [8]. In the present study, a functional analysis of the ammonium transporter in *S*. *mutans* was performed. When the Amt proteins from *Saccharomyces cerevisiae* [45] and *Arabidopsis thaliana* [46] were first described, it was confirmed that the ability of those bacterial cells to take up ammonium may not be a consequence of simple diffusion across the cell membrane, but may also be mediated by an integral membrane protein under some conditions. NrgA deficiency in *S. mutans* was found to result in a delay in growth in medium supplemented with ammonium chloride, urea, calcium chloride, and manganese sulfate. Further, growth rate data demonstrated that NrgA is required for transport and utilization of ammonium in *S. mutans*, which is involved in transport of other inorganic metabolites used as nutrient sources. The physical and chemical properties of ammonium determine its interactions with transporters that are specific for it, as well as other channels that transport ions and molecules. The K^+^ and H_2_O channels have been suggested to have a relationship in transport of ammonium in *A*. *thaliana* and *S*. *cerevisiae* [47]. It has also been speculated that if other channels are involved with the transport of ammonium, then the presence of an ammonium transporter may be relevant not only to the transport of ammonium, but also for other inorganic metabolites. These may be important for uptake of essential nutrients and maintenance of cellular ionic homeostasis as well as for growth.

Adherence to abiotic surfaces and subsequent biofilm formation are two primary steps in the etiology of caries formation by *S. mutans* strains [48]. This process is mediated by glucans and promotes tight adherence and coherence of bacterial cells bound to each other and abiotic surfaces, leading to formation of microcolonies of *S. mutans* and thereby modulating the initial steps of cariogenic biofilm development [49]. When glutamine was added, biofilms formed by NRGD had a coarse structure with reduced biofilm mass. Therefore, it is suggested that inactivation of the ammonium transporter gene is possibly responsible for the non-responsiveness of NRGD to the presence of glutamine, which resulted in a reduction in biofilm formation. On the other hand, in the presence of 20 mM ammonium chloride, the growth of NRGD slightly changed as compared to that of the wild-type at pH 7.0, but was clearly delayed as compared to that of the wild type at pH 5.0. These results suggest that the ammonium transporter may be sensitive to acidic conditions. The GlnR regulon has been identified in *S. mutans* [50] and has a strong correlation with the expression of genes encoding proteins involved in glutamine and glutamate metabolism in response to acid stress [51]. Acidic conditions affect the ammonium transporter more than neutral conditions [10]. In addition, transported glutamine can be converted to glutamate and ammonia by the action of an intracellular glutaminase as an important source for nitrogen and this system is also pH-sensitive [52]. Furthermore, the growth of NRGD in the presence of 20 mM urea was delayed compared to the wild-type, increasing the concentration of urea to 40 mM restored the growth of NRGD (Fig. S2). These results suggested that the growth of NRGD recovered by metabolizing alternative nitrogen source. Together, these results indicate that the *nrgA* gene products play important roles in *S. mutans* biofilm formation, possibly by modulating *nrgA* expression in response to specific environmental conditions.

Genes encoding ammonium transporters have been reported for several bacterial species including *Azopirillum brasilense* [53], *Corynebacterium glutamicum* [54], and *Lactococcus lactis* [55]. Enteric bacteria such as *E. coli* express a specific ammonium or methylammonium ion transport system under nitrogen limiting conditions [56]. In *B*. *subtilis*, ammonium utilization is dependent upon the *nrgAB* operon [57]. NrgA is a membrane protein, whereas NrgB is a member of the PII family. The *nrgB* gene closely resembles the *glnB* gene in *S. mutans*. In the present study, the *nrgA* gene of *S. mutans* MT8148 (serotype *c*) was characterized based on a search for a homologue of the ammonium transporter gene in other bacteria at the initial stages of development of a published database. The amino acid alignment of NrgA encoded by the *S. mutans nrgA* gene suggests that those of *L*. *lactis* and *B*. *subtillis* are closely related with 70% and 50% identites, respectively. The similarity between the genes is 60%. This finding suggests that the *nrgA* gene could act as an ammonium transporter in *S*. *mutans*.

It has been reported that bacterial ammonium transporter activity is also regulated by a second gene [4], with the PII proteins GlnB and GlnK the most common, and their expression is usually regulated by nitrogen [58]. Several mechanisms function to control the expression and activity of the glutamate to glutamine pathway for the utilization of secondary nitrogen source in the presence of glutamine [59]. In *B. subtilis*, synthesis of glutamine synthetase is regulated by the repressor GlnR [60]. An important component in signalling of nitrogen supplie in both Gram-negative and Gram-positive bacteria is exemplified by the *E. coli* PII protein, encoded by *glnB*. This small protein can be uridylated on a tyrosine residue under conditions of nitrogen limitation. A PII-like protein is also present in *E. coli* and in most other prokaryotes. This protein, GlnK, is usually encoded in an operon or gene cluster with an ammonium transporter, AmtB [61]. In *E. coli*, GlnK is also subject to modification by uridylation at low ammonium concentrations. At high ammonium concentrations, free GlnK binds to AmtB and prevents ammonium uptake by this transporter [18]. Therefore, the regulation of nitrogen metabolism is coordinated by the PII type signal transduction proteins. The PII protein family is composed of proteins that regulate enzyme activity, gene expression and are involved in nitrogen regulation, as well as glutamine synthesis activities in bacterial species. In the present study, two lines of evidence suggest that *nrgA* and *glnB* are co-transcribed as a single operon. Further, the *glnB* gene was found to act as a regulator of the *nrgA* gene. Based on real-time RT-PCR assays, it is suggested that interaction between these genes is dependent on the presence of functional NrgA. Deletion of the *nrgA* gene significantly reduced NRGD expression. Therefore, the *glnB* portion of the *nrgA* operon may function as a regulatory gene. Furthermore, the *glnB* gene may act as a receptor and transfer the signal to the *nrgA* gene, suggesting a direct physical interaction between these proteins. Indeed, the location of PII could play a crucial role in regulation of the expression or activity of glutamine synthase (GS) and nitrogenase [13]. Glutamine is synthesized from glutamate and ammonium, a major means for cells to assimilate nitrogen required for biosynthesis [62]. Growth rate data with ammonium chloride in the present study showed decreased growth of NRGD. Therefore, glutamine that is derived ultimately from ammonium was selected as a nutrient for the growth medium in the confocal biofilm and real-time RT-PCR assays. Based on observations of formed biofilms using confocal laser microscopy and a real-time RT-PCR assay, it was concluded that glutamine is a potential inducer of the *nrgA* operon.

The present findings suggest that the *nrgA* gene of *S. mutans* is essential for biofilm formation and export of molecules by the organisms, while the *glnB* gene may regulate *nrgA* expression. In addition, it was shown that biofilm formation occurs in response to the availability of nutrients supplied by the ammonium transporter. Since ammonium transporters are responsible for the movement of ammonium ions across cell membranes and fundamental for nitrogen metabolism in bacteria [9], [63], [64], elucidation of how these transporters are regulated at the genetic level may provide substantial insight into their metabolic pathways. Clearly, further studies are required to provide a better understanding of the role of the ammonium transporter in *S. mutans* in relation to the virulence of this pathogen.

## Supporting Information

Figure S1 **Bacterial growth rates of MT8148, NRGD, and NRGD-comp at pH 5.0 and pH 7.0.** **A and B.** THB only. **C and D.** THB with 20 mM ammonium chloride. **E and F.** THB with 40 mM ammonium chloride.▪ MT8148, ○NRGD, ▴ NRGD-comp. There were significant differences in the values between MT8148 and the two strains (*P<0.05, **P<0.01, and ***P<0.001, ANOVA).
(PPTX)

Figure S2 **Bacterial growth rates of MT8148, NRGD, and NRGD-comp with urea A. THB only.** **B.** THB with 20 mM urea. **C.** THB with 40 mM urea.▪ MT8148, ○ NRGD, ▴ NRGD-comp. There were significant differences in the values between MT8148 and the two strains (*P<0.05, **P<0.01, and ***P<0.001, ANOVA).
(PPTX)
