# Supplementary material for: Identification and Functional Analysis of an Ammonium Transporter in Streptococcus mutans
Source: PLoS One. 2014 Sep 17;9(9):e107569. doi: 10.1371/journal.pone.0107569 (PMC4167856; doi:10.1371/journal.pone.0107569)
Supplement: Figure S1 — Bacterial growth rates of MT8148, NRGD, and NRGD-comp at pH 5.0 and pH 7.0. A and B. THB only. C and D. THB with 20 mM ammonium chloride. E and F. THB with 40 mM ammonium chloride.▪ MT8148, ○NRGD, ▴ NRGD-comp. There were significant differences in the values between MT8148 and the two strains (*P<0.05, **P<0.01, and ***P<0.001, ANOVA). (PPTX) [file pone.0107569.s001.pptx]

## Slide 1
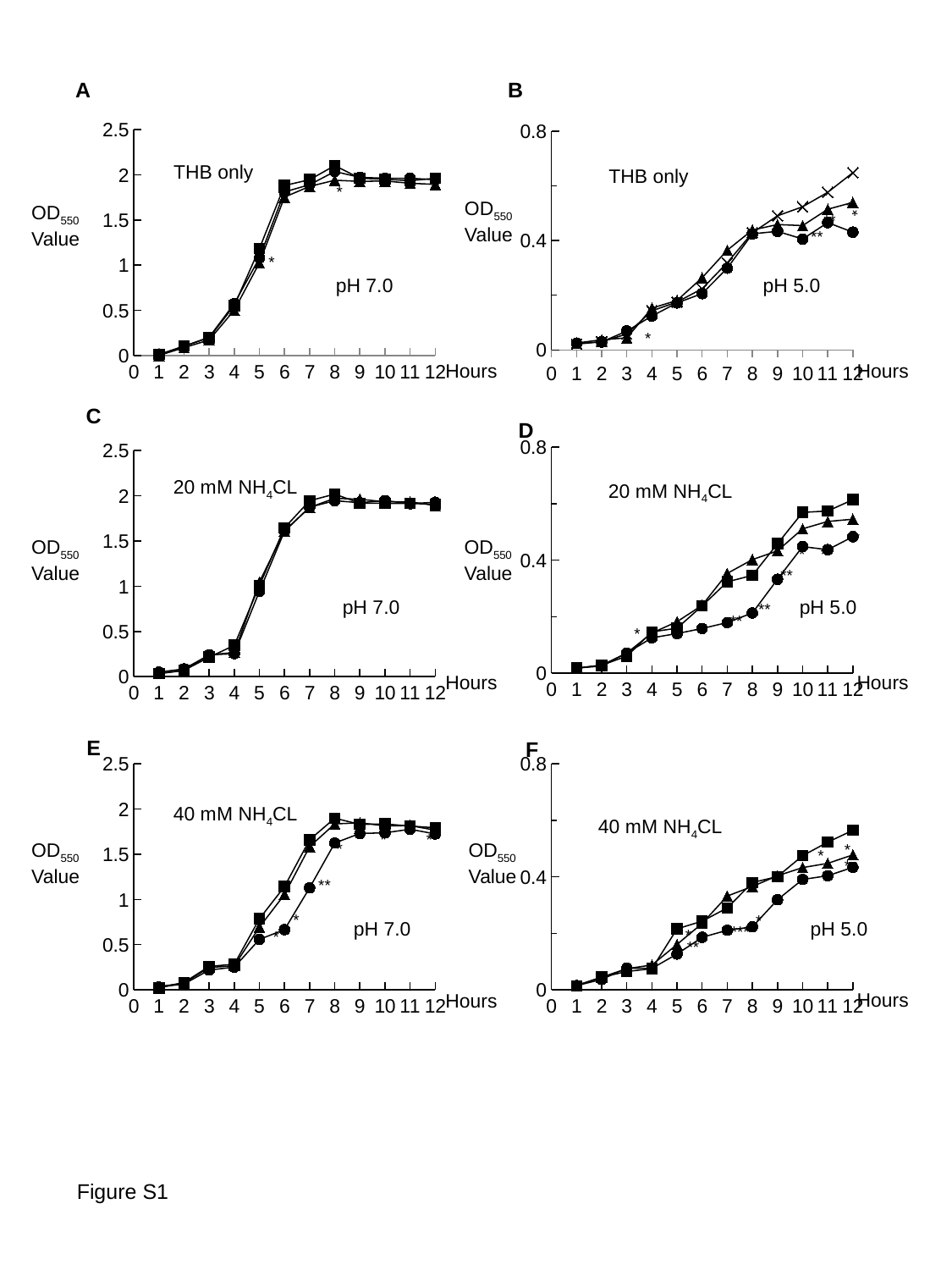

A
B
### Chart
| Category | | | |
|---|---|---|---|
| 0.0 | None | None | None |
| 1.0 | 0.002 | 0.013 | 0.007 |
| 2.0 | 0.089 | 0.106 | 0.103 |
| 3.0 | 0.173 | 0.197 | 0.204 |
| 4.0 | 0.504 | 0.555 | 0.576 |
| 5.0 | 1.026 | 1.184 | 1.08 |
| 6.0 | 1.754 | 1.882 | 1.811 |
| 7.0 | 1.872 | 1.946 | 1.891 |
| 8.0 | 1.94 | 2.102 | 2.037 |
| 9.0 | 1.928 | 1.962 | 1.972 |
| 10.0 | 1.932 | 1.951 | 1.962 |
| 11.0 | 1.907 | 1.935 | 1.962 |
| 12.0 | 1.894 | 1.962 | 1.946 |
### Chart
| Category | | | |
|---|---|---|---|
| 0.0 | None | None | None |
| 1.0 | 0.021 | 0.025 | 0.024 |
| 2.0 | 0.03 | 0.037 | 0.029 |
| 3.0 | 0.058 | 0.044 | 0.069 |
| 4.0 | 0.143 | 0.153 | 0.124 |
| 5.0 | 0.175 | 0.181 | 0.172 |
| 6.0 | 0.224 | 0.264 | 0.206 |
| 7.0 | 0.319 | 0.365 | 0.299 |
| 8.0 | 0.429 | 0.439 | 0.425 |
| 9.0 | 0.491 | 0.459 | 0.434 |
| 10.0 | 0.524 | 0.455 | 0.406 |
| 11.0 | 0.577 | 0.515 | 0.466 |
| 12.0 | 0.649 | 0.54 | 0.431 |THB only
THB only
*
OD550
Value
*
OD550
Value
**
**
**
*
pH 7.0
pH 5.0
*
Hours
Hours
C
D
### Chart
| Category | | | |
|---|---|---|---|
| 0.0 | None | None | None |
| 1.0 | 0.018 | 0.018 | 0.017 |
| 2.0 | 0.028 | 0.025 | 0.028 |
| 3.0 | 0.06 | 0.071 | 0.071 |
| 4.0 | 0.146 | 0.142 | 0.125 |
| 5.0 | 0.159 | 0.182 | 0.14 |
| 6.0 | 0.238 | 0.24 | 0.158 |
| 7.0 | 0.323 | 0.353 | 0.179 |
| 8.0 | 0.346 | 0.402 | 0.213 |
| 9.0 | 0.458 | 0.433 | 0.332 |
| 10.0 | 0.569 | 0.511 | 0.448 |
| 11.0 | 0.574 | 0.537 | 0.437 |
| 12.0 | 0.614 | 0.545 | 0.483 |
### Chart
| Category | | | |
|---|---|---|---|
| 0.0 | None | None | None |
| 1.0 | 0.035 | 0.033 | 0.05 |
| 2.0 | 0.07 | 0.068 | 0.085 |
| 3.0 | 0.215 | 0.241 | 0.24 |
| 4.0 | 0.346 | 0.267 | 0.254 |
| 5.0 | 1.007 | 1.047 | 0.943 |
| 6.0 | 1.646 | 1.612 | 1.609 |
| 7.0 | 1.946 | 1.872 | 1.875 |
| 8.0 | 2.019 | 1.973 | 1.946 |
| 9.0 | 1.919 | 1.962 | 1.924 |
| 10.0 | 1.917 | 1.934 | 1.947 |
| 11.0 | 1.916 | 1.933 | 1.913 |
| 12.0 | 1.907 | 1.893 | 1.929 |20 mM NH4CL
20 mM NH4CL
**
OD550
Value
OD550
Value
*
**
**
pH 7.0
pH 5.0
**
**
*
*
Hours
Hours
E
F
### Chart
| Category | | | |
|---|---|---|---|
| 0.0 | None | None | None |
| 1.0 | 0.022 | 0.036 | 0.031 |
| 2.0 | 0.082 | 0.075 | 0.065 |
| 3.0 | 0.259 | 0.248 | 0.219 |
| 4.0 | 0.284 | 0.266 | 0.252 |
| 5.0 | 0.786 | 0.693 | 0.558 |
| 6.0 | 1.142 | 1.055 | 0.666 |
| 7.0 | 1.661 | 1.585 | 1.129 |
| 8.0 | 1.899 | 1.836 | 1.625 |
| 9.0 | 1.831 | 1.848 | 1.73 |
| 10.0 | 1.835 | 1.815 | 1.738 |
| 11.0 | 1.813 | 1.817 | 1.777 |
| 12.0 | 1.793 | 1.767 | 1.725 |
### Chart
| Category | | | |
|---|---|---|---|
| 0.0 | None | None | None |
| 1.0 | 0.014 | 0.016 | 0.015 |
| 2.0 | 0.045 | 0.045 | 0.037 |
| 3.0 | 0.065 | 0.075 | 0.077 |
| 4.0 | 0.075 | 0.088 | 0.076 |
| 5.0 | 0.216 | 0.161 | 0.127 |
| 6.0 | 0.243 | 0.235 | 0.186 |
| 7.0 | 0.29 | 0.332 | 0.211 |
| 8.0 | 0.38 | 0.366 | 0.223 |
| 9.0 | 0.401 | 0.404 | 0.319 |
| 10.0 | 0.475 | 0.433 | 0.391 |
| 11.0 | 0.523 | 0.448 | 0.404 |
| 12.0 | 0.565 | 0.478 | 0.434 |40 mM NH4CL
40 mM NH4CL
*
*
OD550
Value
OD550
Value
*
*
*
*
*
*
**
*
*
pH 7.0
pH 5.0
***
*
*
**
Hours
Hours
Figure S1
