# Supplementary material for: Identification and Functional Analysis of an Ammonium Transporter in Streptococcus mutans
Source: PLoS One. 2014 Sep 17;9(9):e107569. doi: 10.1371/journal.pone.0107569 (PMC4167856; doi:10.1371/journal.pone.0107569)
Supplement: Figure S2 — Bacterial growth rates of MT8148, NRGD, and NRGD-comp with urea A. THB only. B. THB with 20 mM urea. C. THB with 40 mM urea.▪ MT8148, ○ NRGD, ▴ NRGD-comp. There were significant differences in the values between MT8148 and the two strains (*P<0.05, **P<0.01, and ***P<0.001, ANOVA). (PPTX) [file pone.0107569.s002.pptx]

## Slide 1
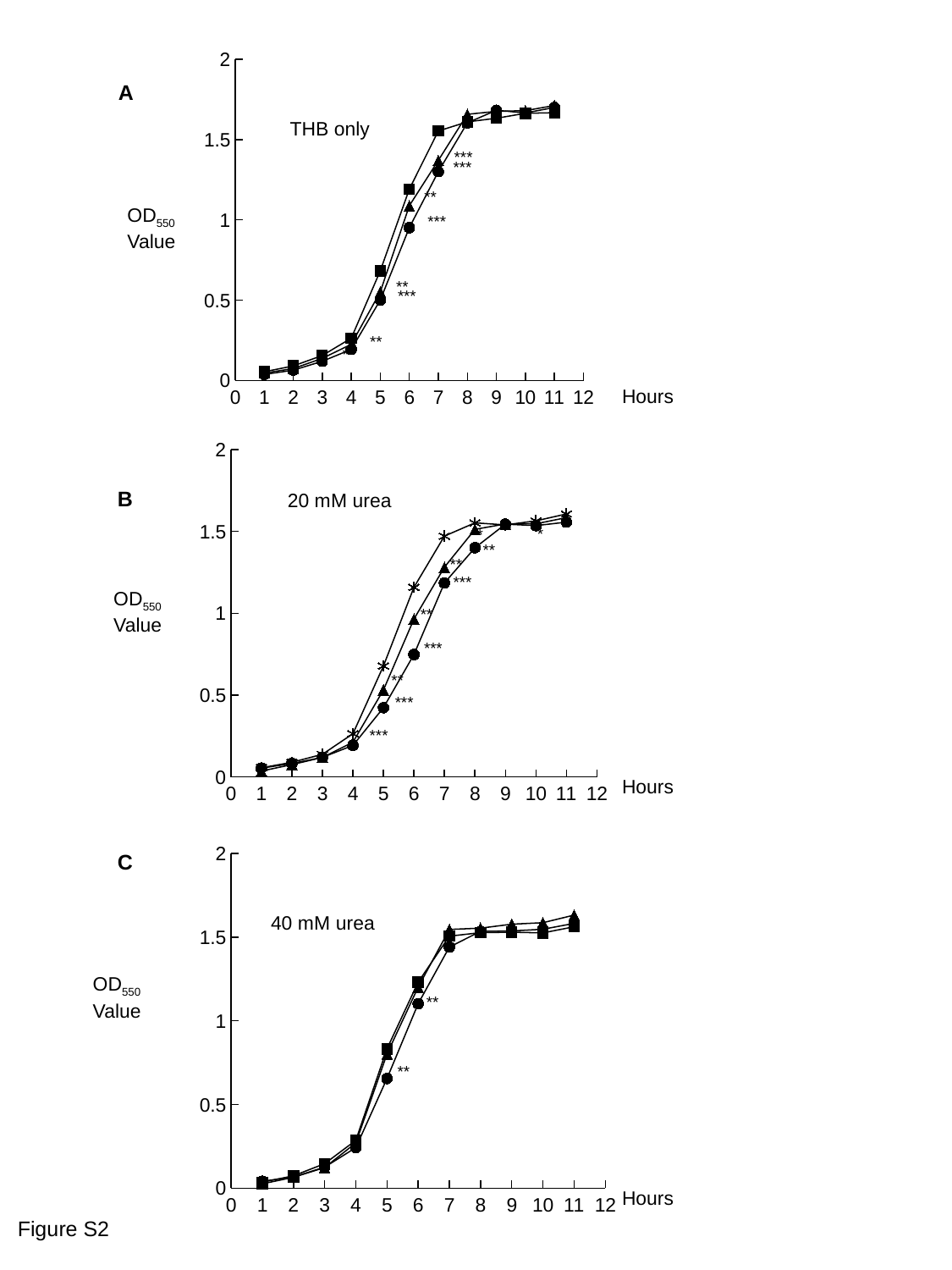

### Chart
| Category | MT8148 | NRGD Comp. | NRGD |
|---|---|---|---|
| 0.0 | None | None | None |
| 1.0 | 0.0538 | 0.0456 | 0.0372 |
| 2.0 | 0.09 | 0.0736 | 0.0638 |
| 3.0 | 0.155 | 0.137 | 0.119 |
| 4.0 | 0.263 | 0.225 | 0.193 |
| 5.0 | 0.682 | 0.551 | 0.5 |
| 6.0 | 1.191 | 1.088 | 0.951 |
| 7.0 | 1.553 | 1.37 | 1.3 |
| 8.0 | 1.61 | 1.657 | 1.604 |
| 9.0 | 1.6324 | 1.6756 | 1.6822 |
| 10.0 | 1.6632 | 1.68 | 1.6656 |
| 11.0 | 1.6672 | 1.7118 | 1.6998 |
| 12.0 | None | None | None |THB only
OD550
Value
Hours
A
***
***
**
***
**
***
**
**
### Chart
| Category | NRGD Comp. | MT8148 | NRGD |
|---|---|---|---|
| 0.0 | None | None | None |
| 1.0 | 0.0362 | 0.056 | 0.054 |
| 2.0 | 0.0756 | 0.0896 | 0.0822 |
| 3.0 | 0.1192 | 0.1386 | 0.12 |
| 4.0 | 0.2126 | 0.2642 | 0.1928 |
| 5.0 | 0.5314 | 0.678 | 0.4232 |
| 6.0 | 0.9647 | 1.1584 | 0.7478 |
| 7.0 | 1.2816 | 1.4716 | 1.1852 |
| 8.0 | 1.5126 | 1.5524 | 1.4002 |
| 9.0 | 1.5458 | 1.5398 | 1.5444 |
| 10.0 | 1.5466 | 1.5648 | 1.5356 |
| 11.0 | 1.5846 | 1.6068 | 1.5566 |
| 12.0 | None | None | None |B
20 mM urea
*
*
**
**
***
OD550
Value
**
***
**
***
***
Hours
### Chart
| Category | NRGD Comp. | MT8148 | NRGD |
|---|---|---|---|
| 0.0 | None | None | None |
| 1.0 | 0.0254 | 0.0352 | 0.0408 |
| 2.0 | 0.0648 | 0.0736 | 0.0664 |
| 3.0 | 0.1228 | 0.1458 | 0.1254 |
| 4.0 | 0.2698 | 0.2854 | 0.2418 |
| 5.0 | 0.8012 | 0.8324 | 0.6548 |
| 6.0 | 1.2014 | 1.2306 | 1.102 |
| 7.0 | 1.5456 | 1.5056 | 1.4404 |
| 8.0 | 1.5534 | 1.5264 | 1.5342 |
| 9.0 | 1.5768 | 1.529 | 1.5366 |
| 10.0 | 1.586 | 1.5248 | 1.5466 |
| 11.0 | 1.6314 | 1.5616 | 1.5816 |
| 12.0 | None | None | None |C
40 mM urea
OD550
Value
**
**
Hours
Figure S2
